# Ferumoxytol Attenuates the Function of MDSCs to Ameliorate LPS-Induced Immunosuppression in Sepsis

**DOI:** 10.1186/s11671-019-3209-2

**Published:** 2019-12-16

**Authors:** Yaxian Xue, Yujun Xu, Xinghan Liu, Zhiheng Sun, Yuchen Pan, Xia Lu, Huaping Liang, Huan Dou, Yayi Hou

**Affiliations:** 1 The State Key Laboratory of Pharmaceutical Biotechnology, Division of Immunology, Medical School, Nanjing University, 22 Hankou Road, Nanjing, 210093 China; 2 The State Key Laboratory of Trauma, Burns and Combined Injury, Research Institute of Surgery, Daping Hospital, The Army Medical University, Chongqing, 400042 China; 3 Jiangsu Key Laboratory of Molecular Medicine, Division of Immunology, Medical School, Nanjing University, Nanjing, 210093 China

**Keywords:** FMT, MDSCs, Macrophages, Sepsis

## Abstract

Sepsis-induced immunosuppression is recognized as one of the main features responsible for therapeutic failures. Myeloid-derived suppressor cells (MDSCs), which are mainly characterized by their suppressive properties, have been reported to be expanded in sepsis. Ferumoxytol (FMT), an FDA-approved iron supplement, has been shown to possess immune-modulatory properties in tumors. However, it is unclear whether FMT alters the functions of MDSCs to reduce late-sepsis immunosuppression. Here, we showed an immunomodulatory effect of FMT on MDSCs to ameliorate lipopolysaccharide (LPS)–induced immunosuppression in the late stage of sepsis. Separation of cells with internalized FMT and detection of the intracellular iron content showed that MDSCs could uptake FMT. Low doses of FMT had no effects on the cell viability of MDSCs, but FMT inhibited the expansion of MDSCs in vitro. Moreover, FMT significantly downregulated the expression levels of Arg-1, S100A8, S100A9, and p47phox as well as ROS production in MDSCs. FMT decreased the percentage of granulocytic MDSCs (G-MDSCs) and promoted the differentiation of MDSCs into macrophages. Furthermore, FMT reduced white blood cell recruitment and alveolar wall thickening in the lungs and areas of necrosis in the liver as well as some biochemical markers of liver dysfunction. FMT decreased the percentage of G-MDSCs and monocytic MDSCs (M-MDSCs) in the spleens of LPS-induced septic mice. Of note, FMT reduced the T cell immunosuppressive functions of both G-MDSCs and M-MDSCs. Expectedly, FMT also significantly reduced Arg-1 and p47phox gene expression in splenic CD11b^+^Gr-1^+^ cells isolated from LPS-challenged mice. These data indicate that FMT decreased the immunosuppressive functions of MDSCs by decreasing Arg-1 and ROS production, suggesting that FMT may reduce long-term immunosuppression in the late stage of sepsis.

## Introduction

FMT, an iron supplement approved by the Food and Drug Administration, has been used for treating iron deficiency anemia in adults with chronic kidney disease (CKD) [1], and FMT is also widely used as a contrast agent and drug carrier [2]. Previous studies showed that FMT had immunomodulatory properties, such as its ability to induce a phenotypic shift in M2 macrophages towards a high CD86^+^, TNF-α positive M1 macrophage subtype [3, 4]. However, the effects of FMT on other immune cells have not been examined.

MDSCs are a heterogeneous population of immature myeloid cells with a potent immune suppressive capacity [5]. In mice, MDSCs are identifiable as Gr-1^+^CD11b^+^ cells, while human MDSCs lack the Gr-1 homolog and are defined as CD14^−^HLA-DR^−^CD11b^+^CD33^+^ or CD14^+^HLA-DR^−^CD11b^+^CD33^+^ cells [6]. MDSCs consist of two large groups of cells, granulocytic or PMN-MDSCs and M-MDSCs, and both populations have immune suppressive functions through the production of iNOS, ROS, and Arg-1. The increased activity of Arg-1 depletes l-arginine, and the shortage of l-arginine inhibits T cell proliferation by decreasing the expression of the CD3 zeta-chain. INOS generates NO, which inhibits the functions of both JAK3 and STAT6 in T cells, as well as the expression of MHC II. ROS induces the posttranslational modification of T cell receptors and may cause antigen-specific T cells unresponsiveness [7]. M-MDSCs arise from monocyte precursors and can differentiate into macrophages and DCs under appropriate cytokine conditions. Most of our knowledge on MDSCs stems from cancer studies. In recent years, an expansion of MDSCs has also been described in acute and chronic inflammatory diseases including inflammation, burns and autoimmune diseases [8, 9]. Recently, a high level of fluorescent nanoparticle uptake by MDSCs in the esophagus and spleen of mice was reported in imaging studies [10]. However, it is unclear whether FMT alters the function of MDSCs involved in the development of inflammatory diseases.

Sepsis, a dysregulated host inflammatory response to severe infection that can cause life-threatening organ dysfunction is the most common cause of death in the intensive care unit. Sepsis initiates an overwhelming proinflammatory response that, if not treated early, rapidly transforms into long-term immunosuppression. The expansion of MDSCs has been described in the spleens and lymph nodes in septic animal models [8, 11], as well as in patients with sepsis [12–14]. The activation of immunosuppressive cells such as MDSCs is critical for appropriate control of the hyperresponsiveness of the innate immune system, but their excess expansion may lead to hyperimmunosuppression, which is the predominant driving force for secondary infection and mortality in late sepsis. Increased numbers of MDSCs correlate with the suppression of lymphocyte proliferation. It has been reported that MDSCs acquire their phenotype in the bone marrow and then migrate to secondary lymphoid organs to inhibit T cell responses and suppress their proliferation in LPS-immunosuppressed mice [8]. Most patients with protracted sepsis exhibit an immunosuppressive status, and most sepsis mortality is due to late-sepsis immunosuppression. Eliminating MDSCs may have beneficial effects in late sepsis by restoring the immune response [15]. Therefore, we hypothesized that FMT may modulate the functions of MDSCs to reduce late-sepsis immunosuppression.

Here we showed an immunomodulatory effect of FMT on MDSCs. We utilized the magnetic properties of FMT to reveal that MDSCs were able to phagocytose FMT in vitro. Moreover, we found that FMT attenuated the immunosuppressive function of MDSCs via downregulation of Arg-1 and ROS. Furthermore, in vivo experiments confirmed that FMT ameliorated the late stage of LPS-induced sepsis in mice by weakening the ability of MDSCs to inhibit T cells. Our data suggest that the immunomodulatory function of FMT may be used to treat the immunosuppression that occurs in late sepsis.

## Materials and Methods

### Mice

Male C57BL/6 mice (8–10 weeks old) were purchased from the Model Animal Research Center of Nanjing University (Nanjing, China). The mice were reared under specific pathogen-free (SPF) conditions on a 24-h light/dark cycle and allowed free access to food and water. All experiments with mice were approved by Nanjing University Institutional Animal Care and Use Committee.

### Prussian Blue Staining

Intracellular FMT distribution was detected using Perl’s Prussian blue staining kit (Solarbio, China) according to the manufacturer’s instructions. Briefly, cells were fixed in 4% paraformaldehyde for 15 min, and then incubated with 10% potassium ferrocyanide for 25–30 min, followed by washing and counterstaining with nuclear fast red for 10 min. Cells containing blue particles in the cytoplasm were positive for Prussian blue staining.

### Generation of Bone Marrow–Derived MDSCs

Bone marrow cells were obtained by flushing the femurs and tibias of wild-type mice followed by RBC lysis. The bone marrow cells were cultured in complete RPMI-1640 medium with 10% FBS, 1% v/v penicillin and streptomycin (Gibco, CA), 40 ng/mL granulocyte-macrophage colony stimulating factor (GM-CSF) (Miltenyi Biotech, Germany) and 40 ng/mL IL-6 (Miltenyi Biotech, Germany) at 37 °C in a 5% CO_2_ incubator for 4 days. Nonadherent cells were collected for further experiments.

### Cell Viability Assay

Cell viability was analyzed using the Cell Counting Kit-8 (Dojino, Kumamoto, Japan) according to the manufacturer’s instructions. Briefly, MDSCs (5 × 10^3^) were plated into 96-well culture plates and incubated in a 5% CO_2_ atmosphere at 37 °C before treatment. The medium was then replaced with fresh medium containing various concentrations of FMT (250, 500, 1000, 2000 μg/mL) for 24 h. The supernatant was subsequently discarded and fresh medium containing CCK-8 solution was added. After incubated at 37 °C for 3 h, the absorbance was measured at 450 nm using a Gen5 microplate reader (BioTek, USA).

### Isolation of Total RNA and Real-time PCR

Total RNA was extracted from cells using the TRIzol reagent (Invitrogen, USA) and quantified with a Nanodrop Spectrophotometer (Thermo Scientific, USA) before being reverse transcribed using the HiScript II 1st strand cDNA Synthesis Kit (Vazyme Biotech Co., Ltd, China) according to the manufacturer’s instructions. Real-time quantitative polymerase chain reaction (PCR) was performed using the SYBR Green PCR Kit (Bio-Rad, Hercules, CA) on an Applied Biosystems StepOne-Plus real-time qPCR system (Applied Biosystems, Forster City, CA). Gene expression was normalized to GAPDH using the 2^−ΔΔCt^ method. The primer sequences are listed in Additional file 1: Table S1.

### Flow Cytometry

Tissues were prepared as single-cell suspensions with collagenase type D (1 mg/mL) and DNase I (0.1 mg/mL) in Hanks’ Balanced Salt Solution (HBSS) at 37 °C for 30 min, and then the red cells of the spleen were lysed. The cell suspensions were filtered through 70-μm cell strainers, and the lymphocytes were collected by centrifugation at 300 g for 5 min at 4 °C. After washing, the cells were immediately prepared for fluorescence-activated cell sorting. Single cells were incubated with FcR blocking reagent (Miltenyi Biotech, Germany) for 10 min, followed by staining with the following antibody conjugates for 20 min: FITC-labeled anti-CD11b, anti-CD11c, anti-CD4 and anti-CD8; PE-labeled anti-Ly6G; APC-labeled anti-Ly6C, anti-CD3, anti-F4/80 and anti-MHC II (BioLegend, CA). After washing, cells were detected with a FACS Calibur (BD, CA) and the data were analyzed using FlowJo 7.6 (Tree Star, Inc., USA)

### H&E staining

Liver and lung tissues were fixed in 4% phosphate-buffered formaldehyde. Fixed tissue was embedded in paraffin, and 5-μm-thick sections were stained with hematoxylin and eosin for light microscopy.

### Enzyme-Linked Immunosorbent Assay

Serum levels of TNFα, MCP-1, and IL-1β were determined using specific ELISA kits (Dakewe, China) according to the manufacturer’s instructions. Each sample was run in duplicate.

### Detection of ROS

Intracellular ROS was measured using the 2,7-dichlorodihydrofluorescein diacetate (DCFH-DA) kit (Beyotime, China ) according to the manufacturer’s instructions. Briefly, the cells were washed twice with PBS and incubated with 5 μM DCFH-DA at 37 °C in the dark for 30 min, then washed with RPMI-1640 medium and resuspended in PBS. Cells were analyzed for intracellular green fluorescence using a flow cytometer (BD, CA).

### T cell Proliferation Assay

Total CD3^+^ T cells were enriched from the spleens of wild-type mice using a CD3ε MicroBead kit (Miltenyi Biotech, Germany) and incubated CFSE stain (Invitrogen, USA). For anti-CD3/CD28 antibody-induced T cell proliferation, T cells were activated with anti-CD3 antibody (1 μg/mL) and anti-CD28 antibody (1 μg/mL). G-MDSCs (Gr-1^high^Ly-6G^+^) and M-MDSCs (Gr-1^dim^Ly-6G^−^) isolated from spleens of FMT-treated or vehicle-treated mice using a Myeloid-Derived Suppressor Cell Isolation kit (Miltenyi Biotech, Germany) according to the manufacturer instructions. MDSCs were cocultured at 1:1 ratio with CFSE-labeled CD3^+^ T cells in 96-well flat-bottom plates for 3 days. CFSE is divided equally among daughter cells with each division; therefore, proliferation was determined by flow cytometry. The purity of the cells after sorting was > 90%.

### Statistics and Data Analysis

All statistics were calculated using the GraphPad Prism 6 software (GraphPad Software, CA) and are presented as the means ± standard deviations (SD). Differences between two groups were analyzed by the Mann–Whitney *U* test or unpaired, two-tailed Student’s *t* test. One-way ANOVA was used for the comparison of multiple groups. All experiments were repeated at least three times. Differences with *p* values < 0.05 were considered statistically significant.

## Results

### A Large Number of MDSCs Uptake FMT

**Fig. 1:**
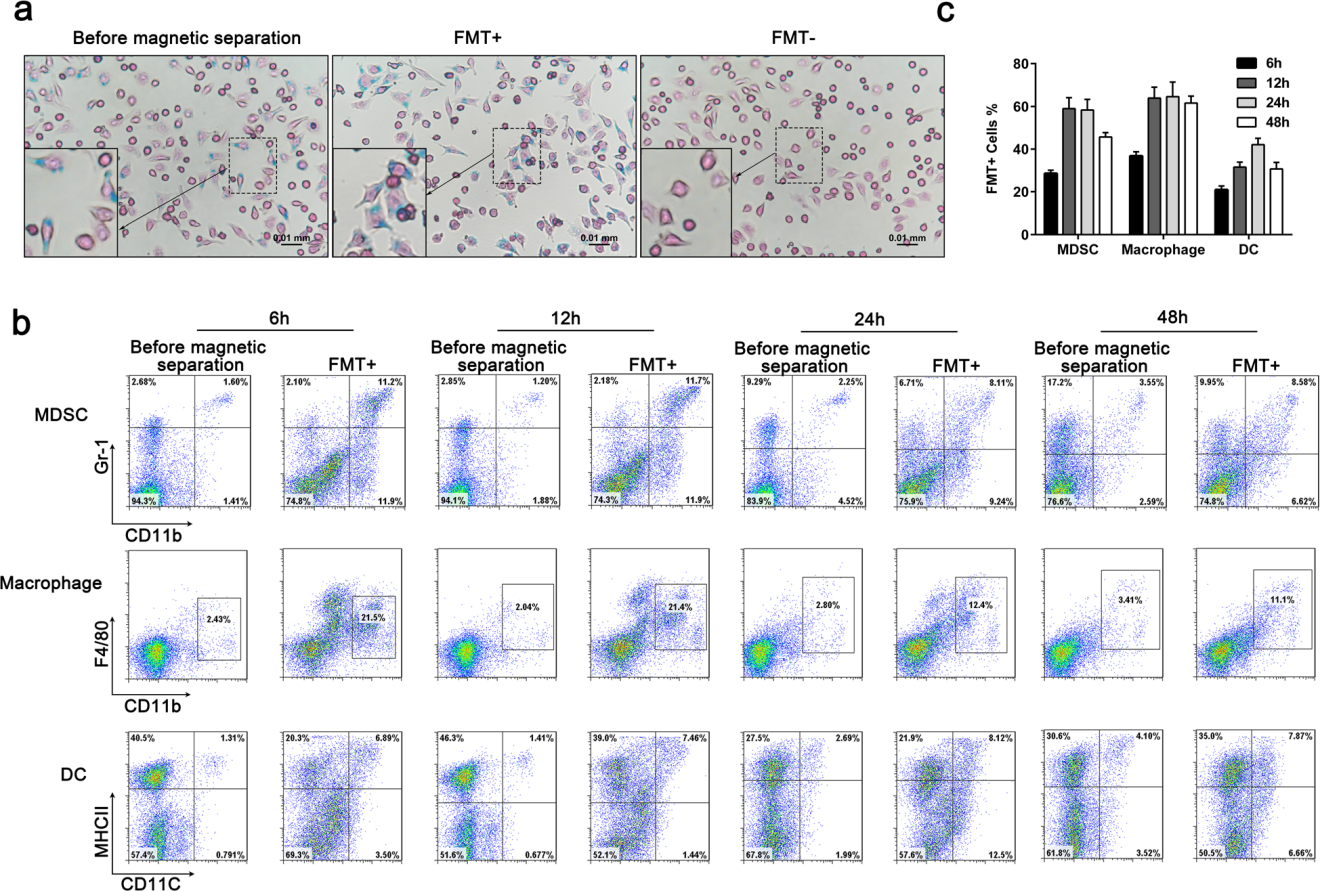
Abilities of MDSCs, macrophages, and DCs to uptake FMT. **a** Macrophages were treated with FMT (1000 ng/mL) for 24 h, then stained with Prussian blue to ascertain the cellular presence and deposition of iron among three groups: before magnetic separation, magnetically selected (FMT+), not magnetically isolated (FMT-). **b** Splenocytes from naïve C57BL/6 mice were incubated with FMT for 6 h, 12 h, 24 h, and 48 h. FACS analysis of the percentages of MDSCs, macrophages, and DCs in two subsets: before magnetic separation, FMT-positive cells. **c** The ratio of FMT-positive cells to cells before magnetic separation. All data are representative of three independent experiments for each experimental group and are displayed as the means ± standard deviation

To verify whether cells with FMT internalized are separated by MACS MicroBeads, we used Prussian blue staining to detect intracellular iron content in macrophages treated with FMT (1000 ng/mL) for 24 h. The cells were divided into three groups: before magnetic separation, FMT-positive cells that were magnetically selected (FMT+), and those that were not magnetically isolated (FMT-). Prussian blue staining demonstrated that the majority of magnetically selected cells were Prussian blue positive (Fig. 1a). To compare the ability to phagocytose FMT between MDSCs, macrophages and DCs, we isolated splenocytes from naive C57BL/6 mice treated with FMT for 6 h, 12 h, 24 h, and 48 h. Splenocytes were divided into two subsets: before magnetic separation and FMT-positive cells. Flow cytometric analysis revealed that nearly 60% of MDSCs and more than 60% of macrophages accumulated FMT after 12–48 h (Fig. 1b, c). Only approximately 40% of DCs were FMT-positive at 24 h. These data showed that like macrophages, MDSCs can uptake FMT and suggested that FMT may influence MDSC function.

### FMT Inhibits the Expansion of MDSCs In Vitro

**Fig. 2:**
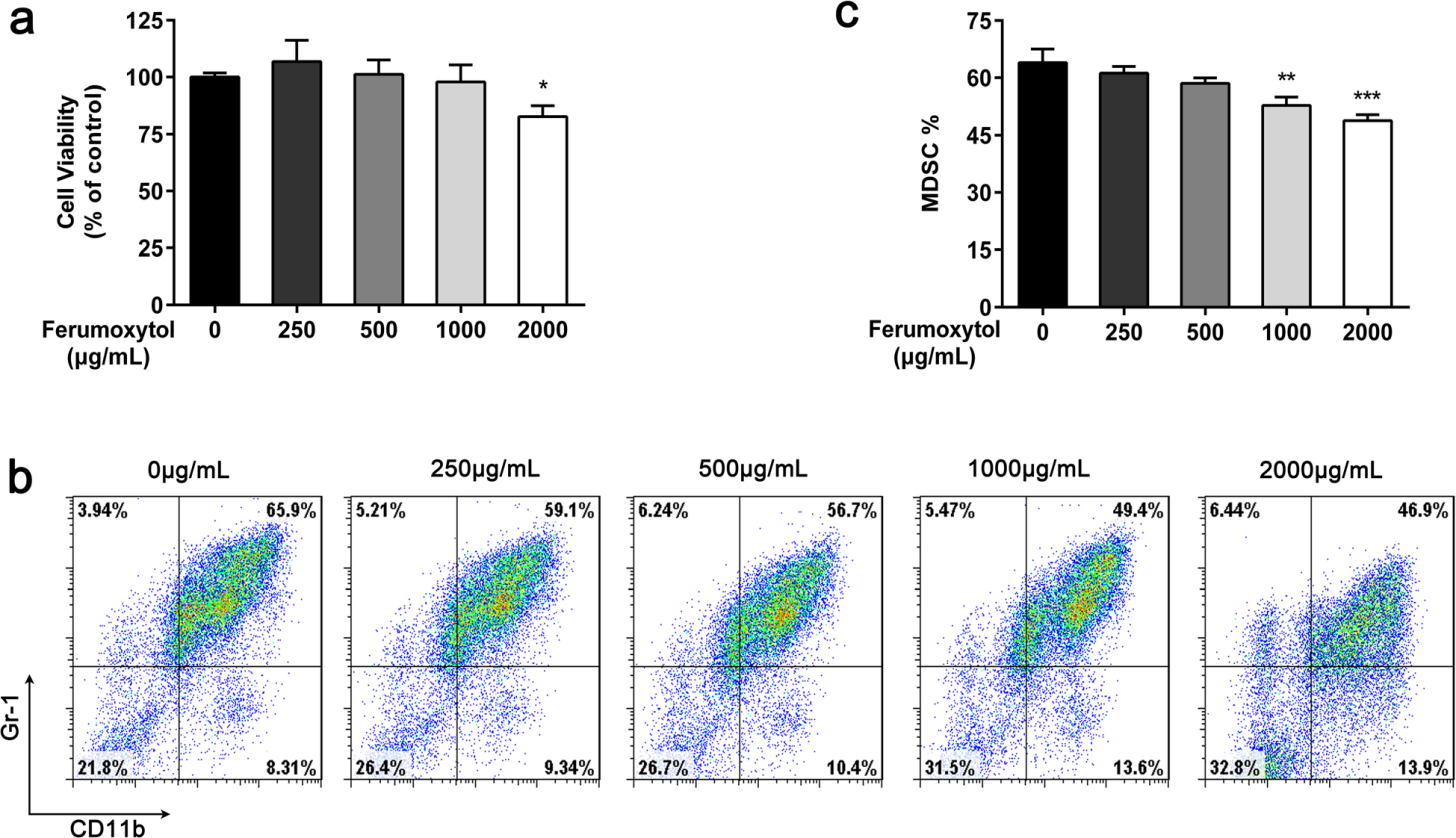
Flow cytometric quantification of the percentage of CD11b^+^Gr-1^+^ cells after treatment with FMT at 250, 500, 1000, 2000 μg/mL in vitro. **a** MDSC cell viability was detected using CCK8 assays after treatment with various concentrations of FMT for 24 h. **b**, **c** Bone marrow cells isolated from C57BL/6 mice were cultured for 4 days with GM-CSF and IL-6 in the presence of different concentrations of FMT, and the cell phenotypes were evaluated by flow cytometry. All data are representative of three independent experiments for each experimental group and are displayed as the means ± standard deviation. *p < 0.05, **p < 0.01, ***p < 0.001 as determined by an unpaired Student’s *t* test versus cells treated with vehicle alone

It has been reported that FMT induces a phenotypic shift in M2 macrophages towards a high CD86^+^, TNF-α positive M1 macrophage subtype [3]. Since there are a large number of MDSCs that can take up FMT, we hypothesized that FMT may alter the function of MDSCs. First, the cytotoxic effects of FMT at 250, 500, 1000, and 2000 μg/mL on MDSCs were evaluated by the CCK8 cell viability assay. The results showed that FMT had no effect on cell viability at low doses and only exhibited moderate cytotoxicity at the maximum dose of 2000 μg/mL (Fig. 2a). We then tested whether FMT at different concentrations would affect the generation of MDSCs. Bone marrow cells isolated from naïve C57BL/6 mice were treated with medium or various concentrations of FMT (250, 500, 1000, and 2000 μg/mL) for 4 days, followed by characterization by flow cytometry on day 4. GM-CSF and IL-6 were added on day 0. The results of three independent experiments revealed that FMT at 1000 and 2000 μg/mL significantly decreased the expansion of MDSCs (Fig. 2b, c).

### FMT Reduces the Immunosuppressive Ability of MDSCs

**Fig. 3:**
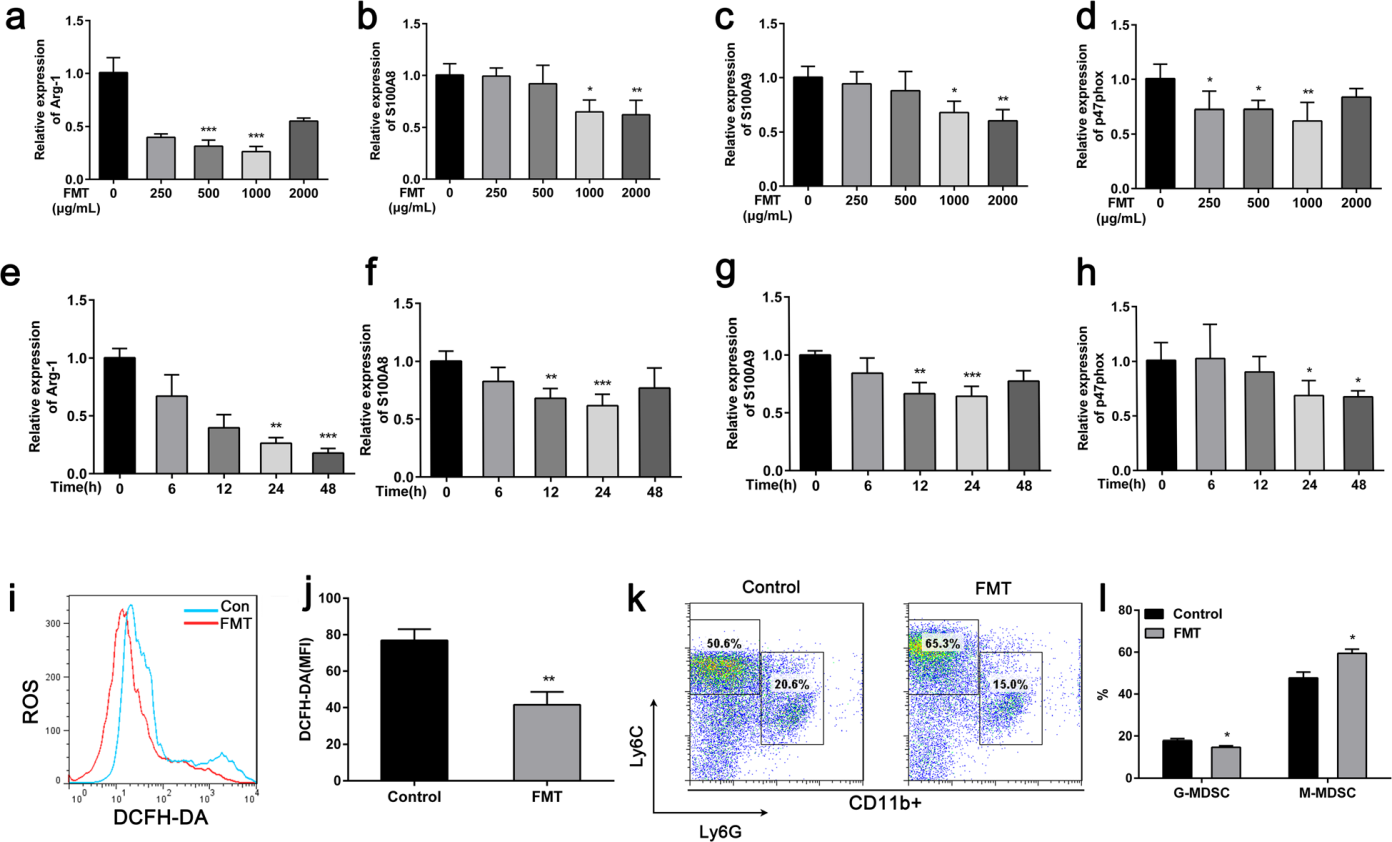
FMT alters the function of MDSCs in vitro. Bone marrow–derived MDSCs treated with vehicle or FMT for 24 h. **a**–**h** Arg-1, S100A8, S100A9, and p47phox gene expression from treated MDSCs as measured by quantitative RT-PCR. **i** BM-derived MDSCs were incubated with FMT for 24 h and ROS production was detected by FCM. **j** Quantitative data of **i**. **k** Subpopulations of MDSCs in the FMT-treated and control groups. **l** FACS analysis of the percentages of G-MDSCs and M-MDSCs. All data are representative of three independent experiments for each experimental group and are displayed as the means ± standard deviation. *p < 0.05, **p < 0.01, ***p < 0.001 as determined by an unpaired Student’s *t* test versus cells treated with vehicle alone

MDSCs use several mechanisms to inhibit the proliferation and effector function of T cells, with the best described being the production of ROS and Arg-1. Expression of the p47phox component of the nicotinamide adenine dinucleotide phosphate–oxidase (NOX) complex is responsible for ROS production in MDSCs [5]. It was reported that S100A8/A9 is synthesized and secreted by MDSCs in an autocrine inflammatory loop and that this is important in the suppression of dendritic cell function in mouse models [16]. To determine if FMT could alter the function of MDSCs, we obtained BM-derived MDSCs as described previously and used RT-PCR to measure mRNA expression in FMT-treated MDSCs versus untreated controls. The results revealed that FMT-treated MDSCs significantly downregulated the expression levels of Arg-1, S100A8, S100A9, and p47phox compared with untreated MDSCs (Fig. 3a–h). Next, we evaluated ROS levels in control MDSCs and FMT-treated MDSCs. The results revealed that FMT-treated MDSCs displayed a significantly lower level of ROS than control cells (Fig. 3i, j). We subsequently compared the changes in the MDSC subpopulations in the FMT-treated and untreated groups. The results showed that FMT decreased the percentage of G-MDSCs, with a slight increase in M-MDSCs also observed (Fig. 3k, l).

### FMT Promotes the Differentiation of MDSCs into Macrophages

**Fig. 4:**
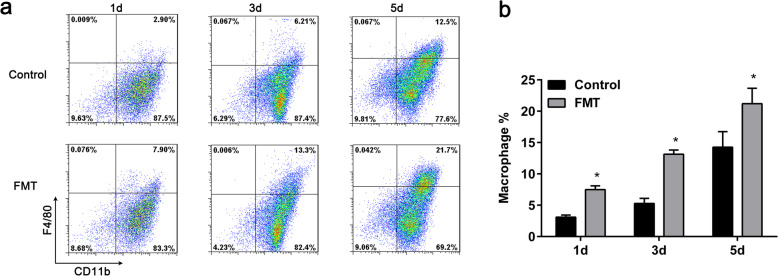
Effect of FMT on the differentiation of MDSCs. **a**, **b** Bone marrow–derived MDSCs were treated with or without 1000 μg/mL FMT and the number of macrophages was assessed after 1 day, 3 days, and 5 days by FACS. All data are representative of three independent experiments for each experimental group and are displayed as the means ± standard deviation. *p < 0.05, versus cells treated with vehicle alone

To determine whether FMT stimulates the final differentiation of MDSCs into macrophages, we treated cells with 1000 μg/mL FMT on day 0 and performed flow cytometry for the macrophage-associated markers CD11b and F4/80 on days 1, 3, and 5. The results showed that 1 day after the addition of 1000 μg/mL FMT the proportion of macrophages increased significantly by more than 2-fold compared with untreated cells. Similarly, 3 days and 5 days after treatment with FMT, the proportions of macrophages were also significantly increased (Fig. 4a, b).

### FMT Ameliorates Symptoms in LPS-Induced Septic Mice

**Fig. 5:**
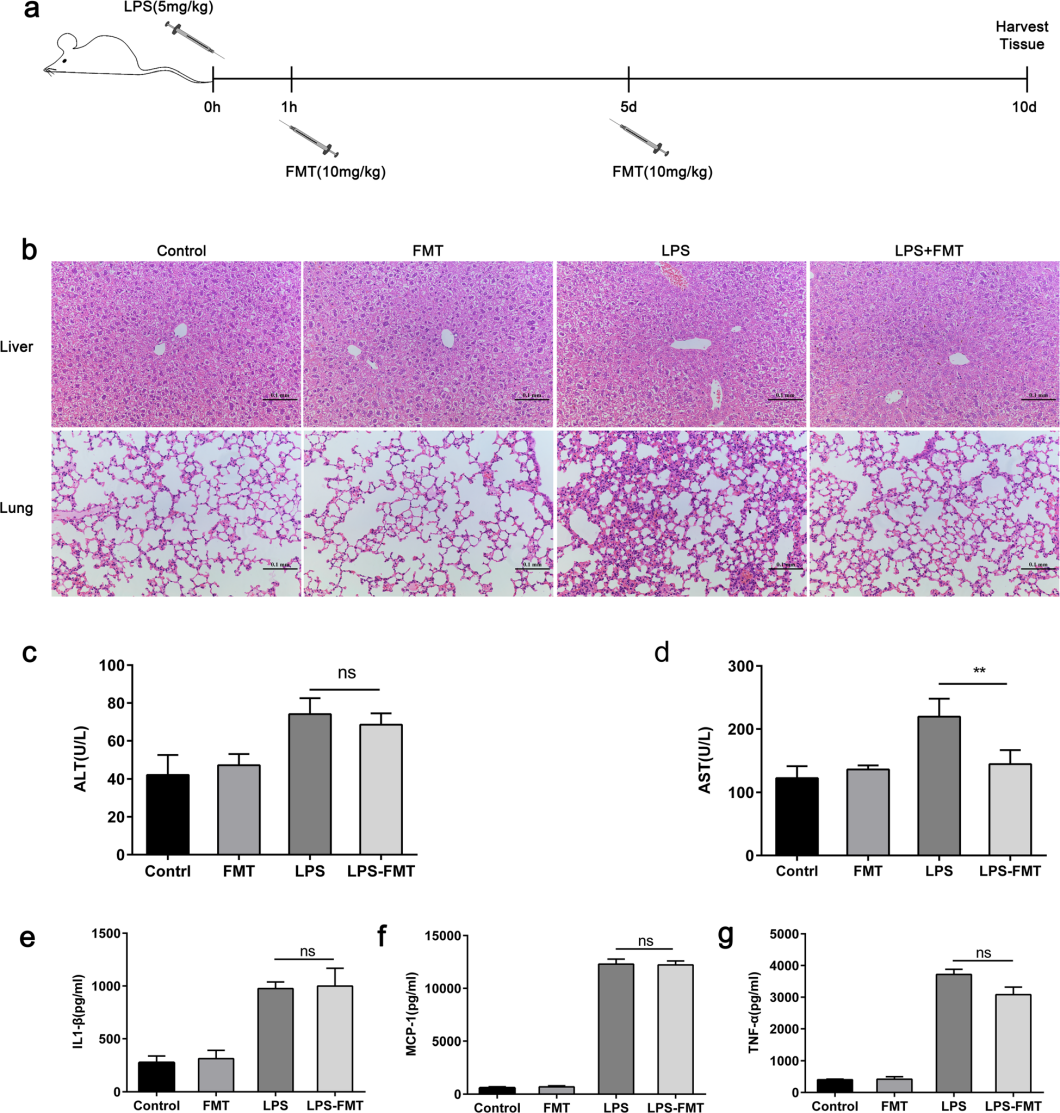
FMT ameliorates liver injury in LPS-induced septic mice. **a** Mice received either PBS or an i.p. injection of 5 mg/kg body weight of bacterial LPS on day 0. After 1 h and 5 days, FMT at a dose of 10 mg/kg or 100 μL saline was administered via tail vein. Mice were divided into four groups (*n* = 6): control, receiving vehicle; LPS, receiving LPS only; FMT, receiving FMT only; and LPS + FMT, receiving LPS and FMT. **b** Lung and liver tissues were stained with hematoxylin and eosin. **c-d** Serum transaminases (ALT and AST) were measured (*n* = 6). Cytokine levels (TNF-a, IL-1β and MCP-1) were measured by ELISA. **e**-**g** Effects of LPS-induced sepsis on cytokine production in vehicle or ferumoxytol-treated mice. Cytokine levels measured by ELISA in the serum of vehicle or ferumoxytol-treated mice following i.p. LPS challenge (50 mg/kg). **p < 0.01 as determined by an unpaired Student’s *t* test

It has been reported that sepsis is associated with tissue damage and leads to organ failure and endothelial dysfunction [17]. To investigate whether FMT reduces late sepsis symptoms, the extent of organ damage in the experimental mice was evaluated after 10 days. A histopathological examination of the lungs in LPS-induced sepsis indicated that FMT reduced white blood cell recruitment and alveolar wall thickening compared with the vehicle-treated mice. H&E staining also revealed that the area of liver necrosis was smaller in the FMT-treated mice compared with the vehicle-treated mice (Fig. 5a, b). Moreover, aspartate transaminase serum levels, used as a biochemical marker of liver dysfunction in these animals, were significantly lower in the livers of FMT-treated mice compared with the control (Fig. 5c). However, FMT did not affect the levels of alanine aminotransferase (Fig. 5d). Furthermore, sepsis is thought to be associated with adverse outcomes such as elevated production of inflammatory cytokines; therefore, we assessed changes in the levels of proinflammatory cytokines in the serum. The level of TNF-α in the serum was slightly, but not significantly, lower in FMT-treated mice at 3 h after LPS injection, and no differences in MCP-1 or IL-1β levels were observed between FMT-treated mice and vehicle-treated mice (Fig. 5e–g).

### FMT Decreases the Number of MDSCs in the Spleen of LPS-Induced Septic Mice

**Fig. 6:**
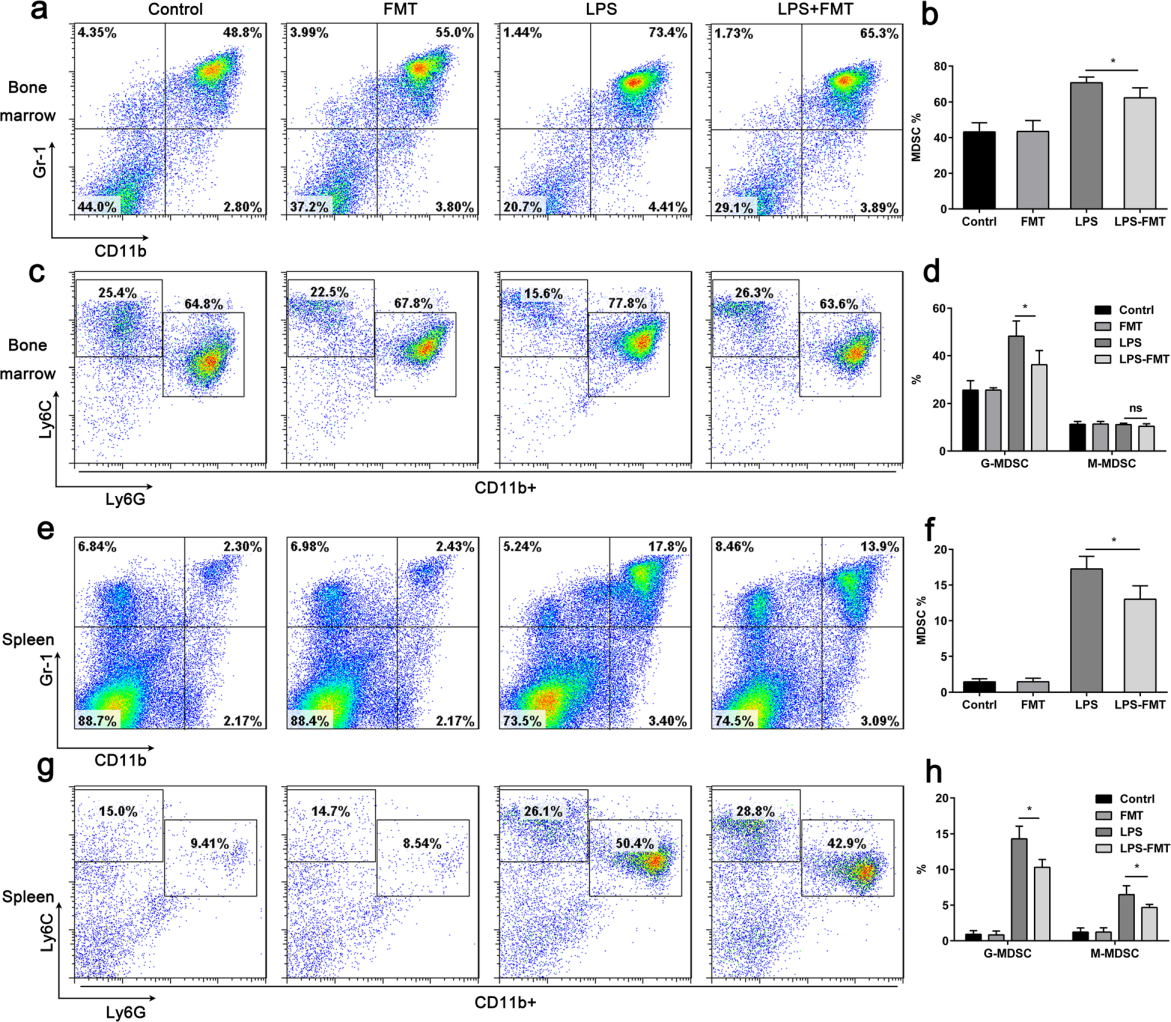
FMT decreases the number of MDSCs in LPS-induced septic mice. Mice were divided into four groups (*n* = 6): control, receiving vehicle; LPS, receiving LPS only; FMT, receiving FMT only; and LPS + FMT, receiving LPS and FMT. **a** CD11b^+^Gr-1^+^ cell populations in the bone marrow were detected by FACS. **b** Quantitative data of **a**. **c** Flow cytometry dot plot of Ly6C and Ly6G expression in the gated CD11b^+^ cells from spleens. **d** Quantification of the percentages of the granulocytic and monocyte subsets. **e** CD11b^+^Gr-1^+^ cell populations in the spleens were detected by FCM. **f** Quantitative data of **e**. **g** Flow cytometry dot plot of Ly6C and Ly6G expression in the gated CD11b+ cells from spleens. **h** Quantification of the percentages of the granulocytic and monocyte subsets. *p < 0.05 as determined by an unpaired Student’s *t* test

Mounting evidence supports that sepsis-associated immunosuppression increases mortality [18], and MDSCs are considered a major component of the immunosuppressive network [8]. It has been reported that G-MDSCs are more specifically expanded in septic patients and appear to be major actors of sepsis-induced immune suppression [12]. To determine whether FMT modulates MDSC populations in wild-type mice, C57BL/6 mice were administered FMT via tail vein on hour 1 and day 5. The results showed that no significant differences in the percentage of MDSCs were detected in the spleen or the bone marrow between the control and FMT-treated mice (Fig. 6a, e). Of note, FMT decreased the LPS-dependent expansion of CD11b^+^Gr-1^+^ cells in both the bone marrow and spleen (Fig. 6a, e), which is consistent with in vitro studies. Moreover, FMT also decreased the percentage of G-MDSCs and M-MDSCs in the spleen, while only the percentage of G-MDSCs was decreased in the bone marrow (Fig. 6c, g). These data indicate that FMT decreases the number of MDSCs in the spleen of LPS-induced septic mice.

### FMT Reduces the T Cell Immunosuppressive Properties of MDSCs In Vivo

**Fig. 7:**
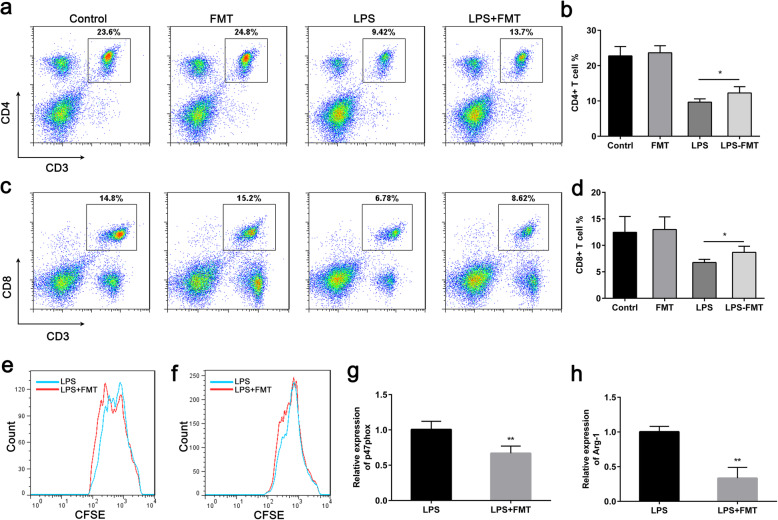
FMT reduces the immunosuppressive properties of MDSCs. **a** CD3^+^CD4^+^ cell populations in spleens were detected by FACS. **b** Quantitative data of **a**. **c** CD3^+^CD8^+^ cell populations in spleens were detected by FACS. **d** Quantitative data of **a**. **e** Ability of G-MDSCs from mice to suppress T cell proliferation induced by anti-CD3/CD28 stimulation was measured by FACS (*n* = 3 per group). A representative histogram is shown. **f** Ability of M-MDSCs from vehicle- or FMT-treated LPS-challenged mice to suppress T cell proliferation induced by anti-CD3/CD28 stimulation was measured by FACS (*n* = 3 per group). A representative histogram is shown. **g**, **h** Arg-1 and p47phox mRNA expression in MDSCs from vehicle or FMT-treated LPS-challenged mice

The numerical reduction of multiple immune cell populations, including CD4 and CD8 T cells, is a major characteristic of the adaptive immune response after sepsis. It is associated with an increased risk of death, as well as other poor outcomes [19, 20]. It has been shown that MDSCs suppress T cell proliferation by impairing T cell zeta-chain expression in sepsis patients [21]. To evaluate the proportion of lymphocytes in late stage sepsis, we measured the proportion of CD4 and CD8 T cells in a murine sepsis model. The results showed that the FMT caused an increase in CD4 and CD8 T cells in the spleens of the LPS-induced mice (Fig. 7a, b). There were no differences in percentages of CD4 and CD8 T cells in the spleen of FMT-treated mice compared with control mice (Fig. 7a, b). To further confirm the role of MDSCs in suppressing T cell proliferation in vivo, G-MDSCs and M-MDSCs were isolated from the spleens of vehicle or FMT-treated LPS-challenged mice by MACS and cocultured with CD3 T cells at a 1:1 ratio. Our results showed that FMT treatment reduced the T cell immunosuppressive functions of both G-MDSCs and M-MDSCs (Fig. 7e, f). Furthermore, we also measured the levels of Arg-1 and p47phox expression in MDSCs isolated from the spleens of mice. As expected, Arg-1 and p47phox gene expression was significantly reduced in splenic CD11b^+^Gr-1^+^ cells isolated from FMT-treated LPS-challenged mice when compared with LPS-challenged mice (Fig. 7g, h). These data indicate that FMT decreased the immunosuppressive functions of MDSCs by decreasing Arg-1 and ROS production, suggesting that FMT may reduce long-term immunosuppression in late-stage sepsis.

## Discussion

The FDA-approved iron supplement FMT has been widely used as a drug carrier and a contrast agent in MRI scanning. It was found that FMT inhibits cancer growth by inducing a proinflammatory immune response with M1 macrophage polarization [3], indicating that FMT has immune-modulatory functions. In this study, we evaluated the effect of FMT on MDSCs by comparing the phenotype and function of FMT-treated MDSCs with control untreated MDSCs. Previous studies have shown that MDSCs are able to take up magnetic nanoparticles [10], and our data further demonstrated that FMT attenuates the immunosuppressive functions of MDSCs in the spleen via downregulation of Arg-1 and ROS. Our data also indicated that FMT has a direct effect on MDSC differentiation, with approximately a fifth of the cells differentiating evenly into macrophages after 5 days in in vitro experiments. Although we did not investigate the phenotype of macrophages exposed to FMT, we hypothesized that they were of the proinflammatory M1 subtype. The results clearly demonstrated that FMT improves the differentiation and maturation of MDSCs, and thus suggests that this underlies the reduction in the MDSC population that we observed during late sepsis.

Sepsis initiates a complex immune response that varies over time and is accompanied by both proinflammatory and anti-inflammatory responses. Previous studies have shown that death in the early stages of sepsis is due to excessive inflammation. Derive and colleagues reported that adoptive transfer of day 10 MDSCs into septic mice attenuated peritoneal cytokine production and improved the survival rate [22]. More recently, Namkoog et al. observed that clarithromycin pretreatment enhanced survival in a mouse model of LPS-induced shock by expanding the CD11b^+^Gr-1^+^ cell population [23]. They described that MDSCs play a protective role in sepsis, however, it should be noted that they were concerned about the early stages of sepsis. Most patients with sepsis display rapid signs of profound immunosuppression, and deaths in this phase are typically due to the acquisition of a secondary hospital-acquired infection, often with opportunistic pathogens [24]. Overzealous MDSC proliferation may facilitate a physiological syndrome of persistent immunosuppression, causing poor outcomes [25]. McClure et al. reported that they did not observe any protective effects from MDSC transfer once the mice entered the late immunosuppressive state [26]. They had previously shown that MDSCs massively expand in the bone marrow, spleen, and lymph nodes of mice with ongoing septic processes and contribute to sepsis-induced T cell suppression [11]. Additionally, Uhel reported that M-MDSCs and G-MDSCs strongly contribute to T cell dysfunction in patients with sepsis [12]. Our data demonstrated that FMT significantly decreased the percentage of MDSCs and attenuated the functions of MDSCs to restore the number of T cells present in mice during late sepsis. Therefore, when studying the role of MDSCs in sepsis, it is crucial to clearly distinguish the early and late stages of sepsis.

Mohus et al. suggested that iron deficiency was associated with increased risk of future bloodstream infections that cause sepsis, indicating that the immune defense mechanisms may be depressed compared to bacterial iron sequestration in low iron environments [27]. Controversies shown in iron supplemental studies reported that intravenous iron therapy was associated with an increased risk of infection [28]. It should be noted the iron supplements differ significantly in their physicochemical properties, which give them different biological properties [29]. With FMT, an iron core is wrapped in a carbohydrate shell, which leads to low toxicity, lysosomal uptake and degradation [30, 31]. It has been reported that FMT does not cause liver toxicity in patients or animal models and is typically metabolized within 2 months [32]. Our data showed that FMT does not increase the production of inflammatory cytokines in early sepsis, indicating that FMT can be administered in sepsis.

## Conclusion

This study demonstrated a novel immune-modulatory property of FMT; however, further studies are needed to elucidate the mechanism of FMT suppression of MDSCs. Furthermore, we provide an attractive therapeutic approach for the treatment of sepsis-associated immunosuppression and targeting MDSCs may provide a promising new option for restoring the immune response during sepsis.

## Supplementary information

**Additional file 1:** **Table S1.** Primers used for real-time quantitative PCR analysis.

## Data Availability

The datasets used and/or analyzed during the current study are available from the corresponding author on reasonable request.

## Abbreviations

ALT: Alanine aminotransferase
Arg1: Arginase 1
AST: Aspartate aminotransferase
FMT: Ferumoxytol
G-MDSCs/PMN-MDSCs: Polymorphonuclear MDSCs/granulocytic MDSCs
HE: Hematoxylin–eosin
IL-1β: Interleukin-1β
iNOS: Inducible nitric oxide synthase
LPS: Lipopolysaccharide
MCP 1: Monocyte chemotactic protein 1
MDSC: Myeloid-derived suppressor cell
MHC II: Major histocompatibility complex
M-MDSCs: Monocytic MDSCs
PBS: Phosphate-buffered saline solution
ROS: Reactive oxygen species
TLR: Toll-like receptor
TNF-α: Tumor necrosis factor α
