# Correction to: Ferumoxytol Attenuates the Function of MDSCs to Ameliorate LPS-Induced Immunosuppression in Sepsis

**DOI:** 10.1186/s11671-021-03624-w

**Published:** 2022-01-04

**Authors:** Yaxian Xue, Yujun Xu, Xinghan Liu, Zhiheng Sun, Yuchen Pan, Xia Lu, Huaping Liang, Huan Dou, Yayi Hou

**Affiliations:** 1 The State Key Laboratosry of Pharmaceutical Biotechnology, Division of Immunology, Medical School, Nanjing University, 22 Hankou Road, Nanjing, 210093 China; 2 The State Key Laboratory of Trauma, Burns and Combined Injury, Research Institute of Surgery, Daping Hospital, The Army Medical University, Chongqing, 400042 China; 3 Jiangsu Key Laboratory of Molecular Medicine, Division of Immunology, Medical School, Nanjing University, Nanjing, 210093 China

## Correction to: Nanoscale Research Letters (2019) 14:379 https://doi.org/10.1186/s11671-019-3209-2

Following publication of the original article [1], it came to our attention that the authorship had been changed without the agreement of all authors prior to publication of the article.

The published article has now been updated with the corrected authorship and the correct authorship may be seen in this erratum.

All authors agree to the updated authorship.

## References

[1] Xu Y, Xue Y, Liu X (2019). Ferumoxytol attenuates the function of MDSCs to ameliorate LPS-induced immunosuppression in sepsis. Nanoscale Res Lett.

