# Supplementary material for: Ferumoxytol Attenuates the Function of MDSCs to Ameliorate LPS-Induced Immunosuppression in Sepsis
Source: Nanoscale Res Lett. 2019 Dec 16;14:379. doi: 10.1186/s11671-019-3209-2 (PMC6915194; doi:10.1186/s11671-019-3209-2)
Supplement: Supplementary file 1 — Additional file 1: Table S1. Primers used for real-time quantitative PCR analysis. [file 11671_2019_3209_MOESM1_ESM.docx]

**Table S1. Primers used for real-time quantitative PCR analysis**

| Gene | Forward | Reverse |
| --- | --- | --- |
| Mouse S100A8 | AAATCACCATGCCCTCTACAAG | CCCACTTTTATCACCATCGCAA |
| Mouse S100A9 | GCACAGTTGGCAACCTTTATG | TGATTGTCCTGGTTTGTGTCC |
| Mouse Arg-1 | CTCCAAGCCAAAGTCCTTAGAG | AGGAGCTGTCATTAGGGACATC |
| Mouse p47phox | ACACCTTCATTCGCCATATTGC | TCGGTGAATTTTCTGTAGACCAC |
| GAPDH | AGGTCGGTGTGAACGGATTTG | GGGGTCGTTGATGGCAACA |
